# NECROTIZING PANCREATITIS: DESCRIPTION OF VIDEOSCOPIC ASSISTED RETROPERITONEAL DEBRIDEMENT (VARD) TECHNIQUE WITH COVERED METALLIC STENT

**DOI:** 10.1590/0102-672020180001e1379

**Published:** 2018-07-02

**Authors:** Eduardo J. HOUGHTON, Alain A. García VÁZQUEZ, Manuel E. ZELEDÓN, Andrea ANDREACCHIO, Gabriel RUIZ, Mariano PALERMO, Mariano E. GIMENEZ

**Affiliations:** 1 Minimally Invasive Surgery, Hospital Bernardino Rivadavia; 2 University of Buenos Aires; 3 DAICIM Foundation, Buenos Aires, Argentina; 4 University of Costa Rica, San Jose, Costa Rica; 5 Percutaneous Surgery, Hospital Piñero, Bueno Aires, Argentina; 6 Hospital Nacional Prof. Alejandro Posadas, Buenos aires, Argentina

**Keywords:** Pancreatitis, Necrotizing Pancreatitis, Videoscopic assisted retroperitoneal debridement, Severe acute pancreatitis, Percutaneous catheter drainage, Step-up approach, Necrosectomy., Pancreatite, Pancreatite necrosante, Desbridamento retroperitoneal videoassistido, Pancreatite aguda grave, Drenagem percutânea, Abordagem gradativa, Necrosectomia.

## Abstract

*****Background*** ****:**

Acute pancreatitis is the third most common gastrointestinal disorder requiring hospitalization in the United States, with annual costs exceeding $2 billions. Severe necrotizing pancreatitis is a life-threatening complication developed in approximately 20% of patients. Its mortality rate range from 15% in patients with sterile necrosis to up 30% in case of infected one associated with multi-organ failure. Less invasive treatment techniques are increasingly being used. These techniques can be performed in a so-called step-up approach.

***Aim*:**

To present the technique for videoscopic assisted retroperitoneal debridement (Vard technique) with covered metallic stent in necrotizing pancreatitis.

***Method*:**

A guide wire was inserted through the previous catheter that was removed in the next step. Afterwards, the tract was dilated over the guide wire. Then, a partially covered metallic stent was deployed. A 30 degrees laparoscopic camera was inserted and the necrosis removed with forceps through the expanded stent under direct vision. Finally, the stent was removed and a new catheter left in place.

*****Result*** ****:**

This technique was used in a 31-year-old man with acute pain in the upper abdomen and diagnosed as acute biliary pancreatitis with infected necrosis. He was treated with percutaneous drains at weeks 3, 6 and 8. Due to partial recovery, a left lateral VARD was performed (incomplete by fixed and adherent tissue) at 8^th^ week. As the patient´s inflammatory response was reactivated, a second VARD attempt was performed in three weeks later. Afterwards, patient showed complete clinical and imaging resolution.

*****Conclusions*** ****:**

Videoassisted retroperitoneal necrosectomy using partially covered metallic stent is a feasible technique for necrotizing pancreatitis.

## RESUMO

***Racional*:**

A pancreatite aguda é a terceira doença gastrointestinal mais comum que requer hospitalização nos Estados Unidos, com custos anuais superiores a $ 2 bilhões. A pancreatite necrosante grave é uma complicação potencialmente fatal, desenvolvida em aproximadamente 20% dos pacientes. A taxa de mortalidade varia de 15% em pacientes com necrose estéril para 30% no caso de uma infecção infectada com falência multiorgânica. As técnicas de tratamento menos invasivas são cada vez mais utilizadas. Elas podem ser realizadas em uma abordagem chamada “step-up”.

***Objetivo*:**

Apresentar a técnica de desbridamento retroperitoneal assistido com videografia (técnica VARD) com stent metálico coberto em pancreatite necrosante.

*****Método***:**

Um fio guia é inserido através do cateter anterior que foi removido no próximo passo. Depois, o trajeto é dilatado sobre o fio guia. Em seguida, um stent metálico parcialmente coberto é implantado. Uma câmera laparoscópica de 30º é inserida e a necrose removida com fórceps através do stent expandido sob visão direta. Finalmente, o stent é removido e um novo cateter deixado no lugar.

***Resultado*:**

Esta técnica foi utilizada em um homem de 31 anos com dor aguda na parte superior do abdome e diagnosticado como pancreatite biliar aguda com necrose infectada. Ele foi tratado com drenos percutâneos nas semanas 3, 6 e 8. Devido à recuperação parcial, realizou-se um VARD lateral esquerdo (incompleto por tecido fixo e aderente) na 8ª semana. À medida que a resposta inflamatória do paciente foi reativada, uma segunda tentativa VARD foi realizada em três semanas mais tarde. Posteriormente, o paciente apresentou resolução clínica e de imagem completa.

*****Conclusão***:**

A necrosectomia retroperitoneal assistida em vídeo com stent metálico parcialmente coberto é uma técnica viável para pancreatite necrosante.

## INTRODUCTION

Acute pancreatitis is the third most common gastrointestinal disorder requiring hospitalization in the United States, with annual costs exceeding $2 billions. Severe necrotizing pancreatitis^1^ ^,^ ^12^ is a common complication developed in approximately 20% of patients. Its mortality rate range from 15% in patients with sterile necrosis up to 30% in case of infected one associated with multi-organ failure^9^ ^,^ ^11^. Less invasive techniques, including percutaneous drainage, endoscopic (trans-gastric) drainage, and minimally invasive retroperitoneal necrosectomy, are increasingly being used^2^ ^,^ ^3^ ^,^ ^4^. These techniques can be performed in a so-called step-up. Compared with open necrosectomy, it can reduce the rate of the composite end point of major complications or death among patients with necrotizing pancreatitis and infected necrotic tissue ^6^ ^,^ ^15^

Initially, the minimaly invasive approach was used for patients not suitable for conventional surgery (conventional necrosectomy has worse outcomes when performed before six weeks of evolution) as a bridge to it. Some of those patients avoided surgery due to their complete clinical resolution even without necrosectomy. On the other hand, patients that continued with inflammatory response not suitable for surgery even beyond 6-8 weeks, were elected to minimally invasive necrosectomy with better outcomes. Therefore, this stepped treatment was born as a need but later became an elective indication, above all, after the PANTER trial^16^. 

This approach utilizes a percutaneous drain or endoscopy to mitigate sepsis^7^ ^,^ ^14^. If drainage fails to control sepsis, the next step is minimally invasive retroperitoneal necrosectomy, videoscopic assisted retroperitoneal debridement (VARD) or sinus tract endoscopy^2^ ^,^ ^3^ ^,^ ^4^ ^,^ ^5^ ^,^ ^8^. 

The objective of this study was to present the videoscopic assisted retroperitoneal debridement (VARD) technique with covered metallic stent in necrotizing pancreatitis.

## METHOD

### Technique

#### *VARD with cystoscope*

**FIGURE 1:**
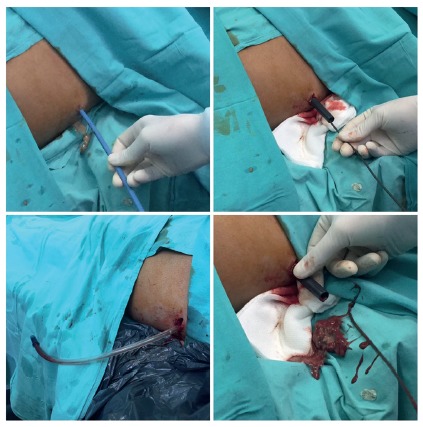
Under fluoroscopic, a guide wire is inserted through the previous catheter which is removed. The tract is dilated increasing size step by step until reach 30 F sheath

**FIGURE 2:**
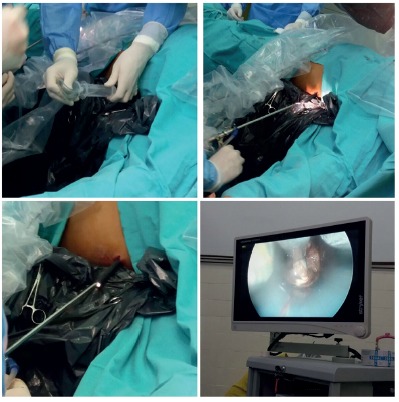
A left lateral video-assisted retroperitoneal debridement was performed. A cystoscope was introduced through the previously described 30 F sheath. An incomplete necrosectomy was performed duo to fixed and adhered tissue.

The first step of this technique is the injection of iodine contrast under fluoroscopy through the percutaneous left lateral drainage to observe the area of necrosis. Secondly, an Amplatz 0,035 guide wire is inserted through the catheter which is removed after. Afterwards, the tract is dilated with progressive plastic dilators to increase the size of the tract to allow a 30 F sheath set in place (Figure 1). Afterwards, a cystoscope is inserted throughout the sheath. Then, the necrosis is removed aslant the working channel of the cystoscope (Figure 2). This procedure is repeated until no more free tissue is found. Finally, a 24 F or similar drainage is left in place. 

#### *VARD with a covered metallic stent*

**FIGURE 3:**
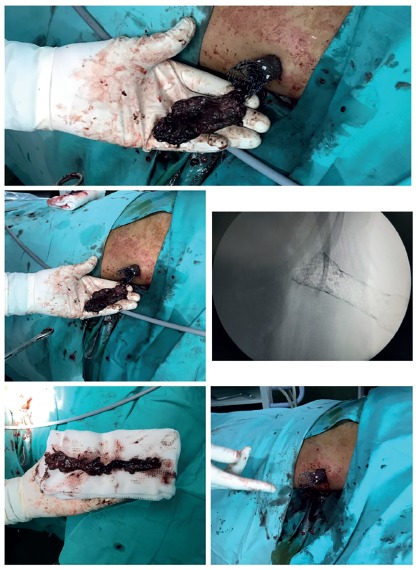
Second VARD using a full covered esophagic metallic stent as a dilatator tract, achieving a complete pancreatic necrosectomy

 The first step of this technique is the injection of iodine contrast under fluoroscopy through the percutaneous left lateral drainage to observe the area of necrosis. Secondly, an Amplatz 0,035 guide wire is inserted through the catheter which is removed after. Afterwards, the tract is dilated with progressive plastic dilators to increase the size of the tract to allow the stent to expand more easily (Figure 1). Once the tract is dilated, the delivery system of a partially covered self expandable metallic stent (with a 22 mm diameter and 9 to 15 cm depending on each patient) is set in place using its radio-opaque marks to leave the distal end at the beginning of the area of necrosis and the proximal end outside the skin. Once the delivery system is correctly set in place, it is deployed (Figure 3). Sometimes, it is necessary to dilate the stent with a 2 cm high-pressure balloon. With the stent completely expanded, a 30 degrees laparoscopic camera is inserted aslant it searching for the pancreatic necrosis. With laparoscopic forceps or curve Foerster forceps and under direct vision of the camera, the necrosis is removed until no more free tissue is found. Finally, a 24 F or similar drainage is left in place. 

## RESULT

**FIGURE 4:**
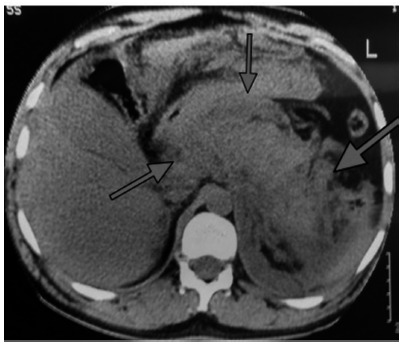
Computed tomography presented acute peri-pancreatic collections and necrosis

This technique was applied in a 31-year-old man presented to the emergency department with acute pain in the upper abdomen. Abdominal ultrasound revealed stones in the gallbladder without dilatation of the bile ducts. Serum laboratory test showed an increasing in serum amylase levels so he was admitted to the surgical ward with the diagnosis of acute biliary pancreatitis. Initial management consisted of fluid resuscitation and analgesics. In the days thereafter the patient appeared to be recovering; however, 1-2 weeks before the admission he deteriorated once more with fever, leukocytosis, an increase in abdominal pain; the contrast enhanced computed tomography presented acute peri-pancreatic collections and necrosis (Figure 4). 

At this time, antibiotics were administered to control the necrosis infection without a clear improvement. Therefore, invasive procedures were attempted in first place: trans-gastric and left lateral percutaneous drains were performed at week 3. Then the trans-gastric drain was replaced at week 6. A second contrast-enhanced CT showed extensive peri-pancreatic necrosis, and for that reason the left retroperitoneal drain was replaced for a larger one. Owing to the subsequent repeated onset of febrile episodes, a left lateral VARD was performed eight weeks of evolution of acute necrotizing pancreatitis (Figures 1 and 2). After tract dilatation using the percutaneous drain as a guide, a cystoscope was introduced through a 30F sheath. Was evacuated some of the free necrotic tissue with instillation of saline water helped by a forceps, leaving one lateral retroperitoneal drain because the necrosectomy was incomplete due to fixed and adhered tissue. After a transient recovery, a relapse occurred and a second VARD was attempted, this time using a partially covered esophagic metallic stent as a dilatator tract, removed inmediatelly after the procedure (Figure 3) and achieving a complete necrosectomy. First a guide wire was inserted through the catheter, then removed. The delivery system of the stent was put in place and then deployed. Afterwards, the necrosectomy was completed using a 30 degrees laparoscopic camera and forceps. 

**FIGURE 5:**
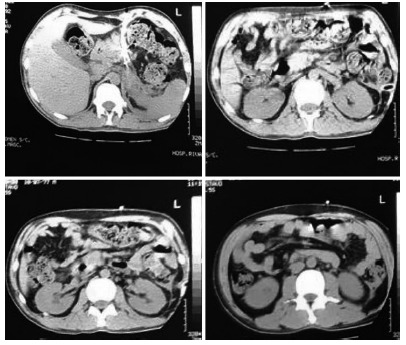
Reduction of peri-pancreatic collection at follow-up CT scan

Patient showed complete clinical resolution and reduction of peri-pancreatic collection at follow-up CT scan (Figure 5). 

The remained peri-pancreatic drains were removed and laparoscopic cholecystectomy was performed in week 12. Three days after the cholecystectomy patient was discharged.

## DISCUSSION

The first step in the surgical step-up approach is antibiotics treatment. Secondly, the placement of a percutaneous drainage (nearly 35-50% of all patients will recover after the first catheter drainage and will not need a necrosectomy) ^13,16^. Depending on the location of the collection, a left- or right-sided, retroperitoneal catheter or both are placed using CT or ultrasonography guidance. The preferred route is through a left-sided retroperitoneal approach^8^, just ventral to the kidney, because when necrosectomy is necessary, this catheter will be used as a guide for VARD without violating the peritoneal cavity. On the other hand, the trans-gastric drainage is recommended to avoid the external fistula. In this case was used 10F drain because the first goal was to gain access to the infected collection, not the necrotic material. These drains are usually flushed with 200 ml of saline three times daily. If there is no clinical improvement, or the patient deteriorates within 72 h after the first drainage, repeat imaging is performed to determine whether these collections are adequately drained. A step-up approach with intention to avoid surgery led to a success rate of 68.5%. Study suggests that a higher percentage of patients can be successfully managed without surgery by an experienced team trained in this approach^10^.

This step-up approach was used with all its phases with an excellent outcome: initially, treatment is done with antibiotics; then percutaneous drainage, re-drainage with larger catheters; afterwards, first VARD (in this case uncompleted due to adhered necrosis) and finally second complete VARD. This strategy reduces the rate of complications and death my minimizing the surgical trauma and the inflammatory response to a surgical intervention.

## CONCLUSION

Videoassisted retroperitoneal necrosectomy using partially covered metallic stent is a feasible technique for necrotizing pancreatitis.
